# Patients’ experiences of discontentment one year after total knee arthroplasty- a qualitative study

**DOI:** 10.1186/s12891-020-3041-y

**Published:** 2020-01-14

**Authors:** Aamir Mahdi, Mia Svantesson, Per Wretenberg, Maria Hälleberg-Nyman

**Affiliations:** 1 Department of Orthopaedics, Faculty of Medicine and Health, Örebro University, Örebro, Sweden; 2 Faculty of Medicine and Health, University Health Care Research Center, Örebro University, Örebro, Sweden; 3 Faculty of Medicine and Health, School of Health Sciences, Örebro University, Örebro, Sweden

**Keywords:** Content analysis, Patient contentment, Patient satisfaction, Qualitative research, Surgery, Total knee arthroplasty

## Abstract

**Background:**

Total knee arthroplasty is a common procedure with generally good results. However, there are still patients who are dissatisfied without known explanation. Satisfaction and dissatisfaction have previously been captured by quantitative designs, but there is a lack of qualitative studies regarding these patients’ experiences. Qualitative knowledge might be useful in creating strategies to decrease the dissatisfaction rate.

**Methods:**

Of the 348 patients who responded to a letter asking if they were satisfied or dissatisfied with their surgery, 61 (18%) reported discontent. After excluding patients with documented complications and those who declined to participate, semi-structured interviews were conducted with 44 patients. The interviews were analyzed according to qualitative content analysis. The purpose was to describe patients’ experiences of discontentment 1 year after total knee arthroplasty.

**Results:**

The patients experienced unfulfilled expectations and needs regarding unresolved and new problems, limited independence, and lacking of relational supports. They were bothered by pain and stiffness, and worried that changes were complications as a result of surgery. They described inability to perform daily activities and valued activities. They also felt a lack of relational supports, and a lack of respect and continuity, support from health care, and information adapted to their needs.

**Conclusion:**

Patient expectation seems to be the major contributing factor in patient discontentment after knee replacement surgery. This qualitative study sheds light on the on the meaning of unfulfilled expectations, in contrast to previous quantitative studies. The elements of unfulfilled expectations need to be dealt with both on the individual staff level and on the organizational level. For instance, increased continuity of healthcare staff and facilities may help to improve patient satisfaction after surgery.

## Background

Total knee arthroplasty (TKA) is a common and effective procedure that is expected to be performed in increasing numbers in the future [1, 2]. Previous studies have shown 6–30% of patients are dissatisfied after the surgery, both in the presence and in the absence of postoperative complications [3–12]. In Sweden, about 8% of patients without documented complications are non-satisfied [13–15].

Quantitative designs have been used to assess patient satisfaction after TKA in previous studies [3, 6–10, 12–31], but to our knowledge no qualitative studies have examined patient’s experience of discontentment after TKA.

The few studies addressing patient satisfaction have focused on the importance of patient education and information before and after surgery [23, 32–37]. One such study showed that patients’ participation in their hospital care facilitated pain management and consequently their satisfaction [36]. Another study of expectations and experiences regarding sports activities among elderly TKA patients concluded that in order for counseling on sports activities after joint replacement to be perceived as supportive, it was important to consider individual motives, barriers, and previous sports experience [4]. 

Similarly, other qualitative studies showed the importance of patient participation, education, and information for satisfaction after TKA surgery [23, 32–35]. Other studies showed that dissatisfaction were correlated with several previous knee surgeries, higher ASA (American Society of Anesthesiologist) score, higher number of comorbidity factors, deprivation and preoperative anxiety/depression [9, 10]. However, these studies are insufficient to describe patients’ experiences of discontentment after TKA surgery.

Orthopedic surgeons have a good awareness about factors affecting patients’ discontentment after TKA [38]. However, several studies showed discordance between the patients’ and surgeons’ satisfactions after TKA [21, 39]. Therefore, we cannot rely our assessments of postoperative patients’ contentment only on orthopedic opinion rather than implying patients’ opinion and experience after surgery.

Capturing knowledge about discontentment can provide complementary knowledge about contentment after TKA, and lead to practical strategies. We therefore aimed to use face-to-face interviews to capture patients’ experiences of discontentment 1 year after TKA surgery without documented complications.

## Methods

The study was approved by the Regional Ethical Review Board of Uppsala, Sweden (ref: 2016/191 [2019–02077]). The participants gave written informed consent, and were informed that participation was on a voluntary basis and that they could withdraw from the study at any time. Confidentiality was guaranteed.

This study had a qualitative approach with an inductive design [40]. All 356 patients who had undergone primary TKA in 2015–2016 at three hospitals in central Sweden were sent a letter 1 year after surgery, asking whether they were “satisfied” or “dissatisfied” with the TKA. Literally, the word “Dissatisfaction” would describe the non-fulfillment of a need or desire [41]. However, most of the participants who answered that they were dissatisfied mentioned that the word dissatisfaction did not describe really their experiences and they got a lot of benefits after TKA. Therefore, we found the word “discontent” more appropriate to describe the participants’ non-satisfaction. Discontentment by definition,” triggered by cognitive stimuli or external forces, is a state of dissatisfaction with one’s circumstances” [42].

**Fig. 1:**
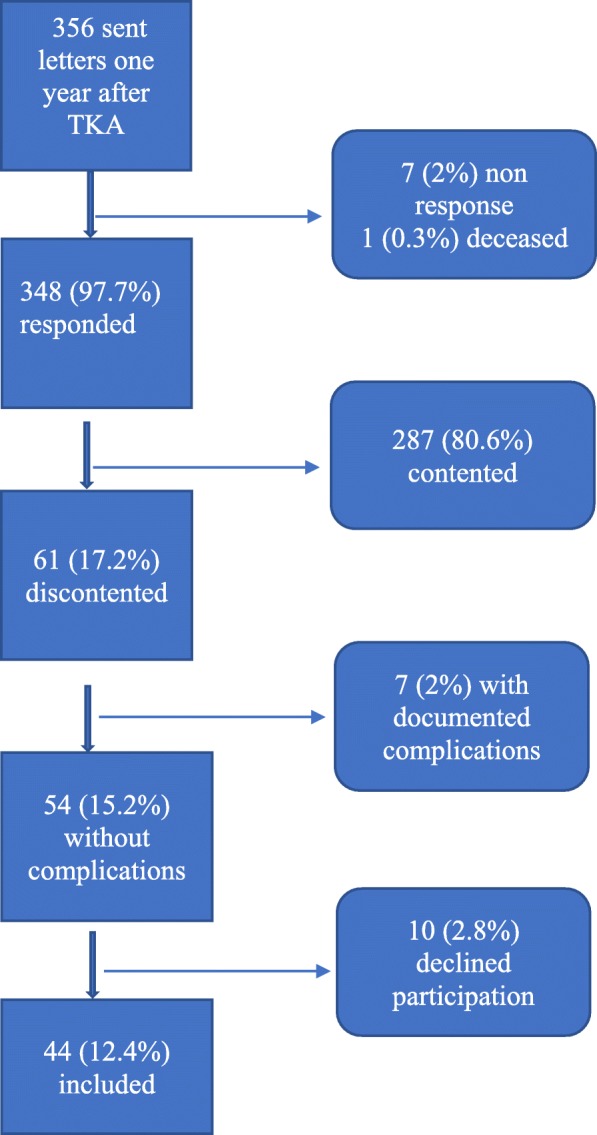
Flow chart showing how the participants were included in the study

Experienced specialist orthopedic surgeons had performed the TKA surgeries using a standard paramedical approach. There were six, four and one orthopedic surgeons working at Hospital 1, 2 and 3 respectively. Each surgeon perform between 25 and 50 TKA per year. Of the 348 who responded, 61 (18%) reported discontentment. Seven patients were excluded due to documented postoperative complications, and a further ten declined to participate for logistical reasons such as difficulty coming to the interview, illness, or late response, and so the final sample consisted of 44 persons (Fig. 1). Out of the final sample, 31(70.5%) participants undergone TKA surgery for the first time while the remaining 13 (29.5%) had undergone TKA previously on the other knee. Table 1 shows an overview of the demographic and clinical characteristics of the study participants.

**Table 1:** Demographic and clinical data for TKA patients

Variable	TKA (discontented) (*n* = 44)	SD	CI	TKA (total) (*n* = 356)	SD	CI	*p*-value
Gender, n (%)							0.83
Female	28 (63.6)			218 (61.2)			
Male	16 (36.4)			138 (38.8)			
Age, range (mean)							0.47
Female	59–88 (70.2)	6.8	66–71	46–91 (70)	8.2	68–70	
Male	59–78 (69.9)	6.4	66–72	48–84 (71)	8.0	66–69	
BMI, mean	28.3	4.2	26.5–30	29.5	4.5	29–30	
ASA, n (%)							0.77
I	(12)26			(77)21			
II	(26)60			(224)63			
III	(6)14			(55)15			
Background, n (%)							0.55
Swedish	41 (93)			341 (96)			
Non-Swedish	3 (7)			15 (4)			
Hospital, n (%)							0.93
Hospital 1	25 (56.8)			203 (57)			
Hospital 2	16 (36.3)			130 (36.5)			
Hospital 3	3 (6.8)			23 (6.5)			
Type of prosthesis, n (%)							0.81
Genesis II TKA	25 (56.8)			201 (56.5)			
NexGen, cemented TKA	18 (41)			141 (39.8)			
NexGen, non-cemented TKA	1 (2.2)			11 (3.1)			
Journey	0 (0)			2 (0.6)			
Side, n (%)							0.19
Right	26 (59)			182(52)			
Left	18 (41)			173(48)			

*TKA* Total knee arthroplasty, *SD* Standard deviation, *CI* 95% confidence interval, *BMI* Body mass index, *ASA* American society of anesthesiologist score (I = normal health, II = mild systemic disease, III = severe systemic disease), Genesis II TKA (Smith & Nephew), NexGen TKA (Zimmer & Biomet), Journey (Smith & Nephew)

Semi-structured face-to-face interviews were conducted at locations chosen by the participants; either in the hospital (*n* = 41) or at their homes (*n* = 3). The first author (AM), who is an orthopedic surgeon, conducted all but one of the 44 interviews. The last author (MHN) conducted one interview because the participant was the first author’s patient. The two main questions were “*Can you tell me about the time before surgery?” and “Can you tell me about the time after surgery?”*. Follow-up questions such as “*Please tell me more about that,*” or “*Can you give an example?*” were used. The interviews also included questions regarding the given information before and after surgery, patients’ expectations and improvements fields. The interview guide was developed specifically for this study (Additional file 1). Participants regret to surgery were also investigated as it might indicate participant’s discontentment regarding TKA surgery. The percentage presented at the end of the results section.

The audio-recorded interviews varied between 15 and 57 min (mean 35 min) and were transcribed verbatim by a professional secretary. Participants were informed that they could request a copy of their transcribed interview. Two participants requested to read their transcribed interview.

The interviews and transcripts were analyzed by means of qualitative content analysis with an inductive approach [40]. Version 11 of the NVivo software package (Boston, MA, USA) was used to facilitate the categorization. Each interview was listened to while reading the text, and the transcripts were read meticulously. Meaning units that related to the aim of the study were extracted and condensed, and then a code was created for each condensed meaning unit. In the next step, the codes were compared for similarities and variations and sorted into sub-categories. The sub-categories were grouped into generic categories, and finally a main category emerged. Data analysis was initiated by the first author (AM), but the progress of each step in the analysis was scrutinized and discussed between AM and MHN. The analysis was co-assessed by MS and then iteratively revised between these three authors until final agreement was established.

## Results

**Fig. 2:**
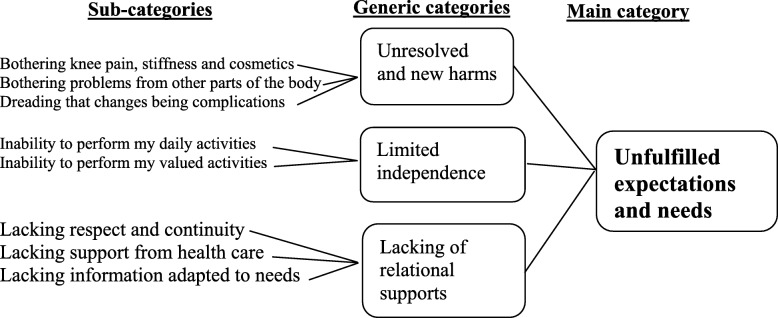
Sub-categories, generic categories and main category describing patients’ experiences of discontentment after TKA surgery

One main category emerged to cover the patients’ experiences of discontentment after TKA: Unfulfilled expectations and needs. The findings are presented in the three generic categories formed in the analysis, all relating to the main category (Fig. 2).

### Unresolved and new problems

Participants experienced unresolved and new problems of bothering knee pain, stiffness and cosmetics, bothering problems from other parts of the body and dreading that changes being complications (Fig. 2).

Pain and stiffness were described either as coexisting in the first three months after surgery or as an isolated complain of either pain or stiffness from the time of performing surgery. Also it could be when the problems did not improve during the first year as expected or a coexisting pain and stiffness during the first three months after TKA which continued as an isolated complain of pain or stiffness after one year from TKA. All of these situations were described by the participants as unexpected and made them discontented. However, the longer the time the participants continued with these complains the more they felt frustration and wondering about the success of surgery.

In the beginning, disappointment was expressed over insufficient pain relief, the fact that medication could not soothe the postoperative pain, and that it did not subside in the first few months. The participants described how disturbed they were with the continual pain, and said that the pain was sometimes so intense that they were unable to do their knee exercises. Some expressed discontentment despite being pain free now because of unpleasant pain experience in the first months after TKA.

*“..But, what happened to me? I didn’t know!..after surgery..after 6 weaks, I could not walk because of high intensity of pain!”* (Participant 1)

Participants expressed disappointment over suffering from existing and new pain as well as the pain having lasted longer than expected.

*“Yeah, it’s been a year. It’s just that …I’ve had this goal the whole time. I’ve complained a bit, and then he’d [doctor] say that it’s only been this and that. Yeah, yeah, Okay. But now it’s been a year., but it is annoying. Because it hurts and I feel,…mm, the longer time I used it the more pain it cause!”* (Participant 19)

Despite the preoperative information that pointed out the likely extent and duration of the postoperative pain, which could be expected to last up to 1 year from the index surgery, some were discontented due to having experienced continued pain for a long time. Pain at night and at rest was described as uncomfortable and worrisome. The participants described discomfort that encompassed the whole knee. The onset of new pain when performing their usual activities and movements created frustration. This kind of pain was described as continuing for several months after surgery, and was experienced as bothersome.

*“ ..It was worst when I was moving..and when I walked in the city, but it was hard to walk so much, because of the pain that I enforced to sit down and take a break!”* (Participant 4)

Discontentment with annoying and long-lasting stiffness was another experience often mentioned by the participants. They expressed their disappointment over not being able to bend and straighten the knee as expected, and said that their knee joint did not feel the same as before. Difficulties getting up from chairs and an inability to kneel for a long time were also mentioned as sources of discontentment.

*“No, straightening it [the knee], that is…the results of the surgery, the range of motion, was not what I had hoped for. And I was disappointed, I still am.”* (Participant 41)

Cosmetic aspects were also mentioned as something annoying. Female participants in particular expressed discontentment over having a swollen knee or still being bowlegged.

*“And get a better-looking knee. That’s what he [doctor] promised. Because it was a bit fat. I asked him if I’d get a good-looking knee, absolutely he said. And now it’s twice as fat.”* (Participant 18)

New or aggravated problems from other parts of the body were also a cause of discontentment, including increased back pain, continued hip pain, pain in the other knee, and pain and swelling in the foot. The participants worried that these changes were symptoms of complications.

The longevity of the long-term after-effects caused the participants to worry and dwell on ongoing complications. Complications that occurred during surgery or a consequence of the surgery were dreaded. The participants said that they worried something was wrong because of, for example, long-lasting pain, swelling, stiffness, and clicking sounds. Numbness, including lack of sensation or no sensation at all after surgery, was described as worrying. The participants worried over whether this was a complication or adverse event.

*“Yeah it’s…how should I say…What’s better with the right knee is that I have more feeling…in the left knee, the feeling has disappeared on the outside, below the knee.”* (Participant 10)

Some of the participants thought that a part of their knee prosthesis was sitting wrong, while others described how they thought they had a detached ligament, fracture, infection, or thrombosis, or that the implant had started to loosen. A few were worried because the knee prosthesis was foreign material that might initiate an inflammatory response or develop into cancer. Most of these participants realized the complexity of a second operation and the subsequent greater risk for complications. Some expressed the fear that they would have to permanently use a wheelchair.

*“… it can trigger cancer and such things [inflammation and infection] …the dentist says that if you’ve had a knee operation then you can’t do this and that.”* (Participant 28)

### Limited independence

Participants told about inability to perform my daily activities and inability to perform my valued activities (Fig. 2). They described how their symptoms hindered them from valued activities. They had expected that after surgery these activities would no longer be a problem, or at least be easier.

Difficulties performing normal daily activities included standing to wash dishes, vacuum cleaning, cutting the grass, working in the garden, driving a car, or performing their jobs; symptoms that were mentioned as obstacles in performing these activities were pain, stiffness, swelling, weakness in the leg, and not being able to trust the knee.

*“..I drive a car outside, but inside my house, cleaning the house for example, I should take a break after half an hour, It is very difficult to continue then!”* (Participant 26)

The participants expressed disappointment over not being able to walk reasonable distances without difficulty or pain after surgery, and felt that it was a realistic expectation to be able to walk normally a year after the surgery. However, they did realize the negative impact that running and jumping would have on the artificial knee. They described different degrees of walking difficulties: some needed to use crutches or walking sticks, some had difficulties walking on uneven as well as flat surfaces, and some had to have a rest after 100 or 250 m.

*“One time, I walked with neighbor, it took more than one hour! Walked with crutches and so on...and took pauses, so that…it was also a flat surfaces,..So,”* (Participant 44)

They also expressed disappointment with their inability or difficulty in performing their favorite recreational activities, such as biking, dancing, hunting, fishing, playing golf, skiing, hiking, swimming, picking berries in the forest, and playing with their grandchildren. Every participant had their own favorite recreational activity or activities, and they spoke about how they had looked forward to resuming these activities after their knee replacements. They described these activities as reasonable for their ages, and noted that there were many elderly people who could perform these activities without problems. Their incapacity for doing these activities was experienced as depressing and disappointing.

*“So, I didn’t cycle at all the year before the operation, and not now either. I just can’t. It makes me sad. I used to cycle all the time.”* (Participant 44)

### Lack of relational supports

Lacking respect and continuity, support from health care and information adapted to needs were described (Fig. 2)*.* Participants described experiencing a lack of respectful interaction from the healthcare staff. For instance, they spoke about how the orthopedic surgeons would enumerate how expensive the surgery was or how the patient was to blame for their knee problem because of their obesity. They also pointed out that when the doctor did not introduce self before surgery, the doctor-patient relationship was damaged and insecurity in the relationship ensued.

*“But I don’t think the aftercare was good. He [doctor] didn’t trust me, he didn’t believe me…he was difficult to deal with, to talk to… he’d look at his watch and say soon it’s time…do you understand.”* (Participant 29)

Participants had previously thought that TKA was a major surgery that required several days in the hospital, and were worried over the short hospitalization. They were surprised to be scheduled for surgery on the morning they arrived at the hospital, and said that being discharged on the same day or shortly thereafter caused a sense of insecurity, especially when they were not discharged by the same doctor they had previously seen and who performed the surgery.

*“They planned to send me home at the second day after surgery when I haven’t stand up yet and got 2 liters of blood!”* (Participant 17)

The participants said they thought individuals should have individually-adapted treatment, and were discontented when they believed they were not getting it. They said they were made to feel they were a bother to the doctors.

Lack of continuity was another source of dissatisfaction. The participants explained how having the same doctor all the time was not only important, but a vital part of health care.

They were rather disappointed when they did not meet their doctor after the surgery and/or at the follow-ups. Participants who were operated on by a different surgeon than the one they were familiar with also voiced discontentment. Some described it as degrading and showing lack of respect.

*“Aha, I thought…so he [doctor] didn’t show up at all, that made me feel…am I worth nothing here now.”* (Participant 9)

The participants also expressed the importance of continuity with the physiotherapist and with the level of care. They felt it inappropriate for patients who had undergone such major surgery to be sent back to their local primary care clinic for follow-ups. They recommended that telephone contact and/or a follow-up visit with the same doctor should be standard procedure.

The lack of individualized training was another unfulfilled need. The participants pointed out the importance of professional, organized, motivated, well-informed, and regular physiotherapy, both pre- and post-operatively, and experienced deviations from these principles as a cause of discontentment. Some of them blamed their perceived poor outcome on the physiotherapist.

*“Yes, of course, it’s that with the physiotherapy. I had the feeling that I should have had help from a physiotherapist sooner, while it was new. I just have that feeling; I think it went wrong. I just lay at home, and I should have pushed harder…that’s what I think, it was a bit wrong. It’s because there was no physiotherapist…”* (Participant 6)

The lack of motivation for training before and after surgery was also mentioned as causing the perceived poor results. The participants realized the importance of training during the year after surgery, but they lacked the motivation. However, they blamed others who they felt had the knowledge and responsibility to motivate them.

*“ ..Perhaps a stricter physiotherapist who creates motivation.. So that they really insist that you do your exercises!”* (Participant 32)

The participants wondered if they had fallen between the cracks in the healthcare system. Some of them criticized the lack of cooperation between the different healthcare facilities responsible for patient care before and after surgery. Sometimes there were disagreements among the facilities, which had a negative impact on the patient. The participants particularly stressed the importance of communication and cooperation between the primary care clinic, the orthopedic department, the different specialists, and the social insurance agency. Limited finances or lack of economic support increased the participants’ feelings of insecurity and discontentment. Most of the financial challenges were related to a prolonged or total inability to return to work, particularly when the insurance agency declined additional support. In some cases, patients who were unable to return to their previous long-term work were asked by the insurance agency to get a different type of job; those who found this impossible then experienced financial difficulties. Added to this were the charges incurred from the continued physiotherapy, doctor visits, and medication.

*“…or if a person says that you should do so…then a person must perhaps plan to change careers or…but for another one who’s worked in that branch or that profession for over 40 years…43 years I think. It’s difficult, of course, when a person has two years left, that is. What should I switch to? …”* (Participant 14)

Discontentment arose over a lack of information regarding the surgery and expected recovery. Some of the participants reported they had wanted more information regarding the risks involved with the surgery and other alternatives to the surgery. The information was experienced as too general in scope, and did not address what each individual patient could expect. One area the participants felt needed addressing was the lack of information regarding the individual training program before and after the surgery.

*“Yes, yes, I probably should have had [individual information]. I did get a pamphlet to read, but it doesn’t discuss that sort of stuff. Instead, it was mostly about how a person should move and which movements you should do and such.”* (Participant 5)

The participants described partial or total regret over having had the surgery. Ten of them (22%) totally regretted the surgical procedure, meaning that the incidence of patients who totally regretted TKA surgery in the absence of complications was 2.9% (10/348). These ten, who included both men and women, said that not only were their expectations unfulfilled, but also new problems and ailments had developed and old ones had worsened considerably after the surgery.

*“I regretted it many times actually. Yeah…the pain was much less before the surgery, than now. Yeah… even if I had major pain and was stiff and such, it was less than this pain, much easier than this pain.”* (Participant 1)

*“I had never experienced a such crises previously in my life!..and I went through massive things..but this time I just fell down in a depression when I went around like that.”*

(Participant 8)

Those participants who expressed partial regret after the surgery did not think the aftercare and results were that worthwhile, and concluded that perhaps they should have postponed the surgery a bit longer.

## Discussion

This study reveals that the expectations and needs of these discontented patients were not met after knee replacement surgery. The participants described unresolved and new problems, lacking of relational supports, and limited independence.

It could be argued that the main-category, unfulfilled expectations and needs, is not one category as it contains both beliefs brought into the surgical experience and experiences that have arisen following surgery. But we meant that the category covered both according to the overall sense of discontentment but also according to specific expectations or needs that were fulfilled in the time that had passed since the TKA surgery. There was no subcategory or generic category which could describe the whole experience of one participant’s discontentment. Participants’ descriptions of their discontentment therefore merged into a main category with combined unfulfilled expectations and unmet needs.

Patients’ expectations have previously been shown to play an important role in postoperative satisfaction [8, 9, 19, 22, 24, 28, 29, 31, 37, 43], but these studies do not define the patients’ expectations, nor do they explain how to address them. The studies do agree about using individualized enquiry regarding patient expectations. Greene et al. recommended preoperative patient education, multimodal pain management, and aggressive postoperative rehabilitation to better meet patients’ expectations [8]. In our study all these recommendations were mentioned by participants as sources of discontentment and as improvement fields for TKA results. The participants each had activities that they expected to be able to perform at least as well as, or better than, before the surgery, and were rather disappointed when these expectations were not met. These expectations need to be deeply explored and defined before surgery to increase patient satisfaction rates in the future. This idea is supported by previous studies which concluded that it is not enough to relate satisfaction to expectations of activities in general terms, but that this should be done on a more personal and specific basis [22, 24, 28].

Patient information plays an important role in patient contentment after surgery. Many studies have shown the positive effects of patient education and information given before surgery [17, 18, 35, 36, 44, 45]. The importance of information and lack of information was made clear by the discontented participants in our study. Although the participants said they were content with the information preoperatively, they were not content with it postoperatively. The discontented participants explained that with more individualized and in-depth information, they could perhaps understand what might have gone wrong and led to their physical complaint. Eschalier et al. supported this in their study by showing the positive effect of educational booklet in improving knowledge among patients waiting for TKA [18].

The other obvious type of information that was missing had to do with their recovery. This was mentioned by most of the participants, which could indicate a considerable problem. All the participants admitted they were told that it would take at least a year after the surgery to see the results, but they said they would have preferred more individualized and detailed information. Also, participants in our study mentioned that the information about complications and pain management they were provided was insufficient. This is in line with the findings in a study by Andersson et al. where it was described that preoperative information on pain relief increased patients’ awareness and participation in postoperative care [36].

Many participants expressed relief at the end of the interview when the researcher offered them an opportunity to receive more information and ask questions regarding their TKA. The therapeutic effect of qualitative interviews is also described in other patient groups [46–48].

The participants in our study described discontentment because they were not fully informed regarding the somewhat limited range of motion that could be expected postoperatively. A recent study showed that an improvement of five degrees in the range of motion in the knee after surgery along with an improved walking distance could increase the satisfaction rate by 6–8 times [49]. Other studies also agree with the effect of stiffness on satisfaction [50, 51]. In the current study the patients’ course of recovery was not investigated, only if there were complications or not. So it was not known whether these patients met the range of motion and walking distance improvements reported in the literature [49].

Our discontented participants expressed anxiety and worry over complications. They saw the longevity of symptoms after surgery as a sign of untoward surgical complications. This might explain why they complained about insufficient follow up and lack of information after surgery. This issue needs to be deeply explored to reduce patient anxiety and minimize discontentment. Our participants also experienced anxiety over the short hospitalization after the TKA, as they had had the impression that the surgery was so major that one or 2 days would be insufficient. They had not been sufficiently informed and prepared regarding this preoperatively. However, many studies have shown that “fast track” TKA procedures, with discharge on the day of surgery or the day after, are associated with good satisfaction rates [52–54]. We did not differentiate between fast track and conventional surgeries, so we cannot say if our results regarding the percentage of dissatisfaction differ from these other study populations. However, because those studies were focused on investigating fast track surgeries, preparation of the patients was perhaps more rigorous and therefore yielded less dissatisfaction in that area, which would be reflected in the overall results.

Pain is the most common factor indicating the need for surgery [55], and is suggested to be the most common cause of dissatisfaction after knee arthroplasty [9, 15, 16]. In our study, participants expressed their anxiety about long-lasting pain. The participants considered postoperative pain lasting more than 3 months to be long-term postoperative pain as. Many participants mentioned varying points of time for when the new onset of pain in the knee occurred after the surgery. They explained how this pain had a new character that did not subside after the wound had healed, said that the pain disturbed their sleep and certain activities, and described the pain as disappointing, unexpected, worrying, and bothersome.

Lacking relational support during their journey played an important role in the participants’ contentment. When they experienced disrespectful interactions with the healthcare staff, they felt degraded and insulted. This could occur throughout the process, and it did not seem to make a difference when. Participants mentioned particularly the need of feedback from their surgeons which would explain a lot of their wonderings. The need for good interaction between the patient and healthcare staff to improve satisfaction after surgery is supported in the literature [7, 28, 35]. On the other hand, lack of social support was more pronounced after surgery. Participants experienced the greatest need for physical, psychological, social, and economic supports during the first 6 months after surgery. A lack of understanding from the insurance agency was worsened by poor communication among the responsible healthcare facilities. Consequently, the patients felt vulnerable without support, which led to discontentment even when they improved clinically afterwards. Weinberg et al. highlighted this issue in a study of patients who experienced problems between themselves and different settings or providers [56]. Lacking continuity in the healthcare team was experienced as troubling in many aspects. One of these was that when the surgeon gets to know the patient well they can undoubtedly better explain the issues related to surgery. This is important, because the participants who reported continued pain and suffering worried that something untoward had occurred. Positive patient experiences and satisfaction after lower extremity arthroplasty have been shown to be closely linked to effective interactions between the patients and the healthcare professionals [57]. To our knowledge, no previous study has addressed orthopedic surgeon continuity and patient satisfaction; this could be a useful issue for future research.

### Limitations, strengths, and trustworthiness of the study

A major strength of using a qualitative method is that it allowed us to capture the content of discontentment. This can serve as a basis for or complement to quantitative study designs, since it provides an understanding of the complexity of non-satisfaction after TKA surgery. The results can be transferred to similar situations or patients, since the participants and context are thoroughly described. Credibility was strengthened by using direct quotations from the participants, and having three different researchers code and analyze the parts of the interviews independently. Dependability was enhanced by the use of triangulation in the agreement of coding and analysis, as well as representation of data from three hospitals. Confirmability was ensured by meticulous data analysis by three of the authors (AM, MHN, and MS).

The large number of interviews (*n* = 44) could be argued both as a weakness and as a strength. On one hand, it can be difficult to grasp the whole of such a large material, and the amount of new findings decreases for every interview after about the first 15 [40]. On the other hand, the large amount of information from these interviews can be seen as representative of this group of discontented patients, and hence the findings can be transferred to similar knee replacement populations.

The fact that the first author (AM) was an orthopedic surgeon and conducted all but one of the 44 interviews can also be viewed as both a weakness and a strength. The researcher’s preunderstanding can be a strength in that it means being familiar with the context and knowing what to ask, but it could also be a weakness if the researcher is not able to see beyond their preconceptions. Another weakness might be that the relationship risks becoming more of a doctor-patient relationship than an interviewer-participant relationship, in that the patient might be inhibited by respect. However, the interviewer used a low profile and had a respectful attitude, and he felt that the informants could express discontentment freely. Additionally, he wore everyday clothing and held the interviews outside the orthopedic department.

Excluding patients with postoperative complications can be regarded as a weakness and a strength as well. These seven patients represents 11% of the possible sample and it could be informative to know their experience of discontentment. On the other hand, there is evidence in the literature which showed the relationship between postoperative complications and dissatisfaction [5, 7–10, 22, 43] but there is little knowledge about dissatisfied patients without an obvious complication. We intended therefore to interview patients who were dissatisfied after surgery without obvious documented complications.

## Conclusion

Discontentment after total knee arthroplasty is a complex problem with many intertwining factors. Although the participants shared common wishes and needs after surgery, they had their own expectations and points of view regarding factors that can lead to the success of surgery. These factors need to be explored and addressed with each individual patient. The elements of unfulfilled expectations need to be dealt with both on the level of individual staff and on the organizational level. For instance, increased continuity of healthcare staff and facilities may help to improve patient contentment after TKA surgery. Furthermore, the importance of both preoperative and postoperative patient education should be an area to focus on in future studies, in order to improve patient satisfaction.

Patient contentment is a process that starts with the first contact before surgery and continues until the patient decides it is complete. Further research is needed to quantitatively analyze the discontentment factors that emerged from this study.

## Supplementary information

**Additional file 1:** Interview guide which was developed for this study and used by the main authors.

## Data Availability

The datasets used and/or analysed during the current study are available from the corresponding author on reasonable request.

## Abbreviations

TKA: Total knee arthroplasty, Genesis II TKA (Smith & Nephew), NexGen TKA (Zimmer & Biomet), Journey TKA (Smith & Nephew)
BMI: body mass index
ASA: American society of anesthesiology
