# Supplementary material for: Patients’ experiences of discontentment one year after total knee arthroplasty- a qualitative study
Source: BMC Musculoskelet Disord. 2020 Jan 14;21:29. doi: 10.1186/s12891-020-3041-y (PMC6961288; doi:10.1186/s12891-020-3041-y)
Supplement: Supplementary file 1 — Additional file 1: Interview guide which was developed for this study and used by the main authors. [file 12891_2020_3041_MOESM1_ESM.docx]

Interview guide –English version

Patients’ experiences of discontentment one year after total knee arthroplasty- A qualitative study

Questions:

1. *Can you tell me about the time before surgery?”*

- Follow-up questions: “*Please tell me more about that,*” or “*Can you give an example? What do you mean? Would you like to explain more? Can you please elaborate more about that? What did you think? Explain more?*
- Please, tell me about your expectations for the knee surgery?
- In what way (to what extent) have these expectations been met or not met?

1. *Can you tell me about the time after surgery?”*

- Follow-up questions: “*Please tell me more about that,*” or “*Can you give an example? What do you mean? Would you like to explain more? Can you please elaborate more about that? What did you think? Explain more?*
- Do you regret the knee surgery? Please tell me why/why not.
- If you were to wish freely, which improvements do you suggest to optimize your experience and the results of your knee replacement surgery?
